# 分散固相萃取-高效液相色谱-串联质谱法测定畜禽肝脏中氟虫腈及其代谢物

**DOI:** 10.3724/SP.J.1123.2021.04007

**Published:** 2022-02-08

**Authors:** Yunji WEI, Huimei BAO, Zhenghe HE, Gaoming HUANG, Min FENG, Zhenyi ZHU, Jian HE

**Affiliations:** 1.淮安海关, 国家饲料安全检测重点实验室(淮安), 江苏 淮安 223001; 1. State Key Laboratory of Feed Safety Testing (Huai'an), Huai'an Customs, Huai'an 223001, China; 2.江苏食品药品职业技术学院, 江苏 淮安 223003; 2. Jiangsu Food & Pharmaceutical Science College, Huai’an 223003, China

**Keywords:** 高效液相色谱-串联质谱, 分散固相萃取, 氟虫腈及其代谢物, 畜禽肝脏, high performance liquid chromatography-tandem mass spectrometry (HPLC-MS/MS), dispersive solid phase extraction (dSPE), fipronil and its metabolites, livestock and poultry liver

## Abstract

欧洲鸡蛋污染事件爆发后,氟虫腈及其代谢物氟甲腈、氟虫腈砜和氟虫腈亚砜残留受到人们较多关注。该研究通过优化前处理方法和色谱条件,建立了畜禽肝脏中氟虫腈及其代谢物的高效液相色谱-串联质谱测定分析方法。样品经10 mL乙腈提取,150 mg PSA、100 mg C18填料净化后,将提取液直接进样分析,以乙腈和水为流动相进行梯度洗脱,在Agilent ZORBAX SB-C18色谱柱(150 mm×2.1 mm, 3.5 μm)上进行色谱分离,采用电喷雾离子源,负离子扫描,多反应监测(MRM)模式测定,基质校正曲线,外标法定量。结果表明,氟虫腈及其代谢物在0.1~10 μg/L范围内线性关系良好,相关系数(*r*^2^)均大于0.995,方法的检出限和定量限分别为0.2 μg/kg和0.5 μg/kg。在0.5、1.0、10.0 μg/kg 3个加标水平下,氟虫腈及其代谢物的平均回收率为81.1%~99.8%,相对标准偏差(RSD)为6.1%~11.7%。应用建立的方法对99份畜禽肝脏样品进行检测,在4份样品中检测出氟虫腈砜,测定值为1.25~2.82 μg/kg。该方法简单,灵敏度和准确度高,适用于大批量畜禽肝脏样品中氟虫腈及其代谢物残留的日常测定。

## Abstract (en)

The residues of fipronil and its metabolites, namely fipronil-desulfinyl, fipronil-sulfone, and fipronil-sulfide, have attracted increasing attention since the European egg contamination incident. In this study, by optimizing the pretreatment method and chromatographic conditions, a simple extraction method coupled with high performance liquid chromatography-tandem mass spectrometry was developed for the simultaneous determination of fipronil and its metabolites, including fipronil-desulfinyl, fipronil-sulfone, and fipronil-sulfide, in livestock and poultry liver. The optimal pretreatment method for fipronil and its metabolites was determined by comparing the recoveries obtained with different extraction solvents (methanol, acetonitrile, acetone, and ethyl acetate), and by purification with *N*-propyl ethylenediamine (PSA) and octadecylsilane (C18). The samples were extracted with 10 mL acetonitrile, then purified with 150 mg PSA and 100 mg C18, following which the extracted solutions were directly injected for analysis. Separation was performed on an Agilent ZORBAX SB-C18 column (150 mm×2.1 mm, 3.5 μm) with gradient elution using acetonitrile and water as the mobile phases. The target compounds were detected by electrospray ionization (ESI) in the negative mode under multiple reaction monitoring, and quantified by the external standard method with matrix-matched standard correction curves. The results indicated that the linear ranges for the four compounds ranged from 0.1 μg/L to 10.0 μg/L with correlation coefficients (*r*^2^) higher than 0.995. The limit of detection was 0.2 μg/kg and limit of quantitation was 0.5 μg/kg. The average recoveries were between 81.1% and 99.8% at three spiked levels of 0.5, 1.0, and 10 μg/kg, with relative standard deviations (RSDs) of 6.1%-11.7%. The matrix effect experiment showed that fipronil and its metabolites had matrix inhibition effects. Matrix-matched standard curve correction was performed to eliminate matrix inhibition effects. The proposed method was used for the analysis of 99 liver samples, where fipronil-sulfone was detected in four samples with values of 1.25-2.82 μg/kg. The method is simple, sensitive, and accurate, and is suitable for the determination of fipronil and its metabolites in livestock and poultry liver.

氟虫腈,商品名锐劲特,是法国罗纳普朗克公司开发的一种含氟、氯的苯基吡唑类广谱性杀虫剂,对农作物上的蚜虫、鞘翅目、蝇类等害虫有很高的杀虫活性,与现有杀虫剂无交互抗性,所以被广泛应用于农作物。由于氟虫腈也可杀灭虱子、跳蚤、螨虫等寄生虫,因此被作为杀虫剂用于畜禽养殖场的卫生消毒。残留在饲料、水、环境中的氟虫腈通过食物链进入鸡体内,导致了荷兰“毒鸡蛋”事件的发生。研究表明,短期大量摄食含氟虫腈食品会对神经系统造成损害,长期摄食含氟虫腈食品会损害肝脏、肾脏、甲状腺,甚至会损害生殖系统^[1]^,氟虫腈对虾类、蟹类等水生生物和蜜蜂有较高的风险^[2]^。氟虫腈在环境中和动物体内不稳定,会代谢成稳定性和毒性更强的氟甲腈、氟虫腈砜、氟虫腈亚砜3种主要代谢产物,欧盟已经明令禁止氟虫腈用于畜禽养殖过程中,规定食品最大限量为0.005 mg/kg,国际食品法典委员会规定动物源性食品中氟虫腈及其代谢物最大限量为0.02 mg/kg^[3]^;我国农业部1157号公告规定,自2009年10月1日起,除卫生用、玉米等部分旱田种子包衣剂外禁止使用氟虫腈,GB 2763-2019《食品中农药最大残留限量》对不同产品中的氟虫腈及其代谢物也制定了详细的最大残留限量^[4]^。

食品中氟虫腈及其代谢物残留量的测定方法主要有气相色谱法^[5,6,7]^、液相色谱法^[8,9]^、气相色谱-质谱法^[10,11,12,13,14]^、液相色谱-质谱法^[15,16,17,18]^,这些方法主要针对植物源性的水果、蔬菜等样品和动物源性的鸡蛋及鸡肉,特别是“毒鸡蛋”事件发生后,鸡蛋、鸡肉中氟虫腈及其代谢物残留量成为人们关注的焦点,检测方法已有大量的文献报道;而对于畜禽肝脏中氟虫腈及其代谢物残留量的分析方法报道较少。实验结合液相色谱-串联质谱联用(HPLC-MS/MS)灵敏度高,抗干扰能力强的特点,在已有文献^[19,20,21]^研究基础上优化前处理方法,对提取溶剂、分散固相萃取填料的种类及用量进行考察,建立了畜禽肝脏中氟虫腈及其代谢物残留量测定的QuEChERS-HPLC-MS/MS方法。

## 1 实验部分

### 1.1 仪器与试剂

Scientific Accela液相色谱仪、TSQ Quantum Ultra EMR质谱仪(美国Thermo公司); ZORBAX SB-C18色谱柱(150 mm×2.1 mm, 3.5 μm,美国Agilent公司); 3K15低温冷冻离心机(德国); SB-5200DTD超声仪(宁波新芝生物科技股份有限公司); Milli-Q纯水仪(美国Millipore公司)。

氟虫腈、氟甲腈、氟虫腈砜、氟虫腈亚砜标准品(纯度均大于99%)购自德国Dr. Ehrenstorfer公司;乙腈、乙酸铵、甲酸均为色谱纯,购自德国Merck公司;*N*-丙基乙二胺(PSA)、十八烷基硅烷(C18)购自上海安谱实验科技有限公司。

### 1.2 标准溶液的配制

标准储备溶液的配制:分别称取适量的氟虫腈、氟甲腈、氟虫腈砜、氟虫腈亚砜标准品,用乙腈溶解,配制成1.0 g/L的标准储备液,于-18 ℃冰箱中避光保存。

混合标准溶液的配制:分别移取适量的标准储备溶液,用乙腈稀释配制成100 mg/L高浓度混合标准溶液,再用乙腈逐级稀释配制成10 mg/L与1 mg/L的混合标准溶液,于4 ℃冰箱中避光保存。

基质标准工作溶液的配制:移取一定体积的混合标准溶液,根据需要用阴性样品提取液稀释成质量浓度为0.1、0.2、0.5、1.0、5.0、10.0 μg/L系列基质标准工作溶液,现用现配。

### 1.3 样品的前处理

准确称取样品5 g(精确至0.01 g),置于50 mL具塞离心管中,加入10 mL乙腈,在涡旋混合器上混匀30 s后,超声提取15 min,以8000 r/min离心5 min,转移全部上清液至50 mL具塞离心管中,加入150 mg PSA、100 mg C18,涡旋1 min,以8000 r/min离心5 min,取上清液过0.22 μm滤膜,供液相色谱-串联质谱仪测定。

### 1.4 分析条件

色谱柱:Agilent ZORBAX SB-C18色谱柱(150 mm×2.1 mm, 3.5 μm);柱温:30 ℃;流动相:A为乙腈,B为水;流速:250 μL/min;进样量:25 μL。梯度洗脱程序:0~3.0 min, 20%A; 3.0~4.0 min, 20%A~60%A; 4.0~6.0 min, 60%A~90%A; 6.0~10.5 min, 90%A; 10.5~11.0 min, 90%A~20%A; 11.0~15.0 min, 20%A。

离子源:电喷雾离子(ESI)源,负离子扫描;离子源温度:400 ℃;离子传输管温度:350 ℃;喷雾电压:3000 V;鞘气压力:4.5 MPa;辅助气压力:1 MPa;多反应监测(MRM)方式检测。其他质谱条件见表1。

**表 1:** 氟虫腈及其代谢物的多反应监测模式的质谱参数

Compound	Retention time/min	Ionization mode	Parent ion (m/z)	Daughter ions (m/z)	Collision energies/eV
Fipronil	7.31	ESI^-^	434.8	249.8, 329.8^*^	29, 18
Fipronil-desulfinyl	7.21	ESI^-^	386.8	281.8, 350.8^*^	33, 17
Fipronil-sulfide	7.39	ESI^-^	450.8	281.8^*^, 414.8	29, 19
Fipronil-sulfone	7.36	ESI^-^	418.8	261.8, 382.8^*^	29, 16

* Quantitative ion.

## 2 结果与讨论

### 2.1 质谱条件的优化

将质量浓度为1.0 mg/L的4种化合物标准溶液通过蠕动泵分别直接注入质谱仪中,进行质谱条件的优化。结果发现氟虫腈及其代谢物在正、负离子模式下均有响应,但负离子模式下仪器响应强度和稳定性均优于正离子模式,因为氟虫腈及其代谢物分子结构中均含有氨基,在正离子模式下有一定的响应,但待测化合物的分子结构中含有多个强负电性基团-Cl、-F、-CN,易于吸附电子形成负离子,所以负离子模式的响应远高于正离子模式,故选择负离子模式进行一级质谱扫描,获得[M-H]^-^准分子离子峰作为母离子。再对每个准分子离子峰进行二级质谱扫描,得到相应的碎片离子信息,并优化碰撞能量、碰撞气、辅助气、喷雾电压、透镜补偿电压等二级质谱参数,选择离子丰度最高的碎片离子为定量离子,离子丰度次高的碎片离子作为定性离子,优化后的参数见表1。

### 2.2 色谱条件的优化

氟虫腈及其代谢物采用负离子监测模式采集,流动相体系一般为碱性或中性,文献^[22]^采用酸性流动相,本文考察了流动相A相为乙腈、甲醇,B相为水、0.1%(v/v)甲酸溶液、5 mmol/L乙酸铵溶液、5 mmol/L乙酸铵溶液(含0.1%(v/v)甲酸)共8种组合流动相体系下目标物的分离效果、响应强度及峰形。结果表明在上述8种流动相体系下4种目标化合物均出峰,在乙腈-水流动相体系下响应强度和峰形最佳,因此选择乙腈-水作为流动相。

### 2.3 提取溶剂的选择

氟虫腈及其代谢物易溶于甲醇、乙腈、乙酸乙酯、丙酮等有机溶剂,实验采用阴性样品添加1.0 μg/kg混合标准溶液,考察甲醇、乙腈、乙酸乙酯、丙酮的提取效果。结果见图1,乙腈沉淀蛋白的效果及提取效率最强,提取液的颜色最澄清,4种有机溶剂中乙腈作为提取溶剂回收率最高;丙酮、甲醇和乙酸乙酯作为提取溶剂回收率较差,丙酮和乙酸乙酯提取的色素、脂肪等非极性干扰物比较多,不利于后续净化;甲醇提取液浑浊且颜色最深,因此选择乙腈作为提取溶剂。

**图1:**
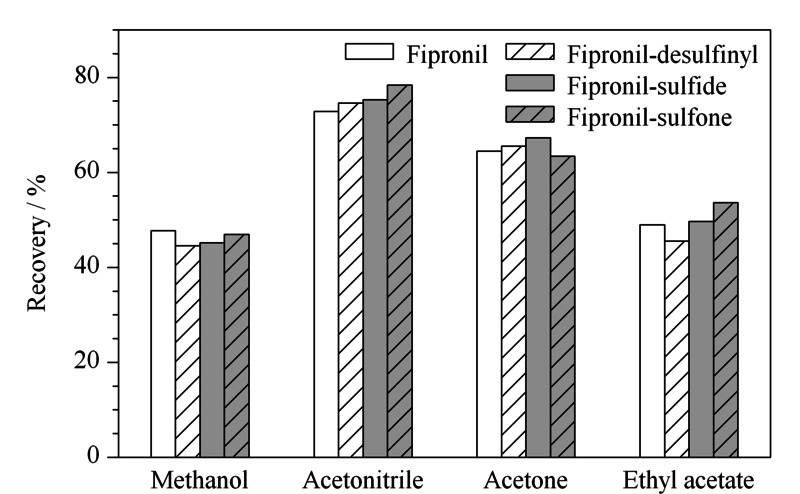
不同提取溶剂对目标物回收率的影响

### 2.4 净化方法的优化

应用QuECHERS方法进行农药残留分析时,常用*N*-丙基乙二胺(PSA)、十八烷基硅烷(C18)、石墨化炭黑(GCB) 3种填料作为吸附剂,PSA能够去除基质中的有机酸、色素、金属离子,C18能够去除脂肪和脂类等弱极性干扰物,GCB主要用于吸附基质中的色素。实验首先考察了3种填料对目标物的吸附性,结果发现GCB对4种目标物的吸附率达31.7%~50.3%, PSA和C18无吸附性,故实验选择PSA和C18两种填料作为吸附剂。实验进一步对填料的用量进行优化,设计了每种填料用量为50、100、150、200 mg 4个质量水平,进行正交实验,结果见图2,综合考虑回收率、经济效益、净化效果,最终净化填料的使用量为150 mg PSA、100 mg C18。

**图2:**
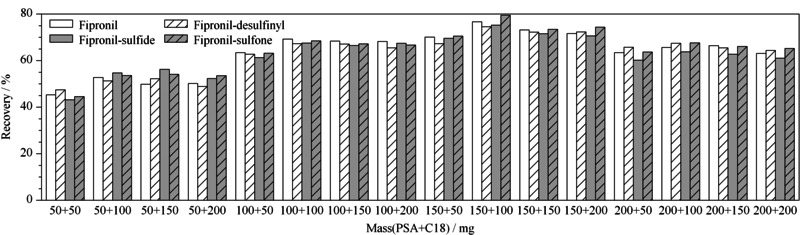
填料用量对目标物回收率的影响

### 2.5 基质效应

采用ESI源分析药物残留时普遍存在基质效应,基质效应影响检测灵敏度、精密度及准确度。消除基质效应的手段主要有优化前处理方法、使用同位素内标、采用基质匹配曲线、减少进样量等。基质效应以公式ME=空白基质标准溶液中氟虫腈及其代谢物的峰面积/溶剂标准溶液中氟虫腈及其代谢物的峰面积×100%计算,当ME大于1时表示基质增强,ME小于1时表示基质抑制。本文采用了梯度洗脱、分散固相萃取填料净化、提取溶液净化后不浓缩直接进样分析等方法,但依然存在基质抑制效应,结果见表2,考虑经济因素,本文采用基质匹配曲线校正回收率,保证测定结果的准确性。

**表 2:** 氟虫腈及其代谢物的线性方程、相关系数、检出限、定量限和基质效应

Compound	Linear equation	r^2^	LOD/(μg/kg)	LOQ/(μg/kg)	ME
Fipronil	Y=251021X+101864	0.9998	0.2	0.5	0.771
Fipronil-desulfinyl	Y=358624X+442629	0.9990	0.2	0.5	0.721
Fipronil-sulfide	Y=442867X+226954	0.9997	0.2	0.5	0.708
Fipronil-sulfone	Y=191301X+233931	0.9983	0.2	0.5	0.720

*Y*: peak area; *X*: mass concentration, μg/L.

### 2.6 线性范围与定量限

取1.2节配制的基质标准工作溶液,按1.4节优化的实验条件进行测定。以分析物峰面积为纵坐标(*Y*)、对应的质量浓度为横坐标(*X*, μg/L)绘制标准曲线,氟虫腈及其代谢物在0.1~10.0 μg/L范围内线性关系良好,相关系数(*r*^2^)均大于0.995;在阴性空白样品中从低到高添加不同水平的混合标准工作溶液,以定量离子对色谱峰的信噪比(*S/N*)大于3和10确定方法的检出限和定量限分别为0.2 μg/kg和0.5 μg/kg,结果见表2。样品中添加0.5 μg/kg目标物的MRM色谱图见图3。

**图3:**
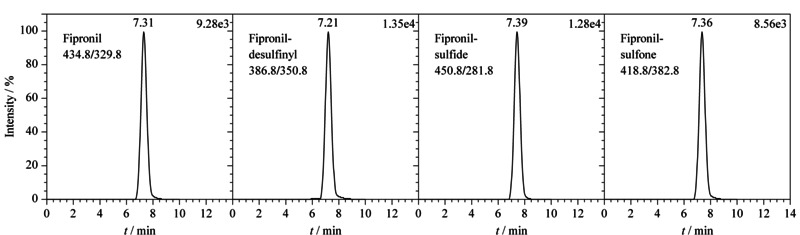
鸡肝中添加0.5 μg/kg氟虫腈及其代谢物的MRM色谱图

### 2.7 回收率及精密度

在阴性空白鸡肝、鸭肝、鹅肝、猪肝4种样品中分别添加0.5、1.0、10 μg/kg 3个水平的混合标准溶液,每个添加水平重复5次试验,用基质匹配曲线校正,外标法定量,得到方法的回收率及精密度,见表3。

**表3:** 畜禽肝脏中氟虫腈及其代谢物的加标回收率和相对标准偏差(*n*=5)

Compound	Added/ (μg/kg)	Chicken liver			Duck liver			Goose liver			Pig liver	
		Recovery/%	RSD/%		Recovery/%	RSD/%		Recovery/%	RSD/%		Recovery/%	RSD/%
Fipronil	0.5	93.5	9.0		98.4	8.9		94.1	10.9		96.7	8.5
	1.0	90.2	7.6		94.4	9.2		99.8	11.2		82.8	10.1
	10	92.6	8.2		87.6	6.1		88.2	8.7		86.5	7.6
Fipronil-desulfinyl	0.5	97.2	9.3		92.5	7.5		93.5	9.2		89.3	8.8
	1.0	95.5	8.5		97.3	8.0		90.4	7.4		95.4	7.2
	10	85.7	7.9		89.6	8.5		97.7	8.9		93.1	9.3
Fipronil-sulfide	0.5	89.6	7.8		82.8	10.6		96.1	11.5		96.2	11.7
	1.0	87.1	6.4		86.4	7.8		86.0	6.7		90.5	9.8
	10	88.5	7.7		91.8	6.3		92.3	9.4		85.6	9.4
Fipronil-sulfone	0.5	92.3	10.1		87.2	8.2		85.7	8.5		93.3	8.3
	1.0	81.1	6.7		91.7	9.6		86.5	7.9		96.5	11.0
	10	86.5	8.5		93.4	7.3		96.2	6.2		85.7	8.6

### 2.8 实际样品检测

用本方法对29份鸡肝、20份鸭肝、15份鹅肝、35份猪肝样品进行测定,仅3份鸡肝、1份鸭肝样品中检测出氟虫腈砜,含量分别为1.73、2.82、2.36、1.25 μg/kg,低于欧盟规定的最大残留限量要求,其余3种化合物均未检出。

## 3 结论

本文建立了高效液相色谱-串联质谱测定畜禽肝脏中氟虫腈及其代谢物残留的方法,优化了前处理方法,提高了灵敏度和准确性,本方法操作快速、简单,稳定性好,检测成本低,方法的检出限、回收率、精密度符合要求,适用于畜禽肝脏中氟虫腈及其代谢物残留检测。
